# Quality indicators for learner-centered postgraduate medical e-learning

**DOI:** 10.5116/ijme.58ce.60aa

**Published:** 2017-04-27

**Authors:** Robert A. de Leeuw, Michiel Westerman, Fedde Scheele

**Affiliations:** 1 VU University Amsterdam, The Athena Institute for Transdisciplinary Research, the Netherlands; 2 VUmc, School of Medical Sciences, Amsterdam, the Netherlands

**Keywords:** Postgraduate medical education, e-learning, distance education, quality, evaluation model, learning theory

## Abstract

**Objectives:**

The objectives of this study were to identify the needs and expectations of learners and educational experts in postgraduate medical e-learning, and to contribute to the current literature.

**Methods:**

We performed four focus-group discussions with e-learning end-users (learners) and didactic experts. The participants were postgraduate learners with varying levels of experience, educational experts from a Dutch e-learning task group, and commercial experts from a Dutch e-learning company. Verbatim transcribed interview recordings were analyzed using King’s template analysis. The initial template was created with reference to recent literature on postgraduate medical e-learning quality indicators. The transcripts were coded, after which the emerging differences in template interpretation were discussed until a consensus was reached within the team.

**Results:**

The final template consisted of three domains of positive e-learning influencers (motivators, learning enhancers, and real-world translation) and three domains of negatively influential parameters (barriers, learning discouragers, and poor preparation). The interpretation of the final template showed three subjects which form the basis of e-learning, namely, Motivate, Learn and Apply.

**Conclusions:**

This study forms a basis for learning in general and could be applied to many educational instruments. Individual characteristics should be adapted to the target audience. Three subjects form the basis of, and six themes cover all items needed for, good (enough) postgraduate e-learning. Further research should be carried out with learners and real-world e-learning to validate this template.

## Introduction

The benefits of medical e-learning courses are well reported and pertain to efficacy, cost-effectiveness, and interactivity.^1^ Furthermore, it is postulated that the number of available courses will continue to increase.^2^ E-learning is described as a viable solution for challenges which range from the promotion of self-directed learning to the provision of flexible learning through the continuous availability of learning opportunities.^3^ Moreover, it engages learners through collaborative learning communities and supports continuous professional development.^3^ These aspects of e-learning could be most beneficial for postgraduate and continuous medical education^4^ however, certain associated downsides must be considered.

In 2014, Cook and colleagues described the myths of e-learning and emphasized the downsides. According to the authors, e-learning is neither cheap, nor inherently more effective or even more efficient, than face-to-face learning.^5^ Neither will it (by itself) transform education. However, the authors described one way in which it might create significant change in medical education (a disruptive innovation): “low-cost, low-tech, and instructionally-sound online learning (‘‘good enough’’ instruction), represents a disruptive innovation that will soon displace high-tech, high-cost, online learning products”.^5^

Sufficient literature exists to support the benefits of e-learning; however, we are also faced with the limitations and “myths”. The difficulty is that there is no clear definition of what constitutes “good enough” instruction. It is not even clear how to measure or define quality in postgraduate e-learning.^6^^,^^7^ A low-fidelity, high-quality approach, with integrated, appropriate learning theory, can successfully overcome many of the barriers to the introduction of e-learning.^8^^,^^9^ A few working models with quality indicators are available, but only one of these is specifically designed for postgraduate learners.^7^ In 2016 we described thirty-seven items in six domains in a quality specifications review, to provide an overview of these quality indicators.^7^ Most of these items are based on expert opinion and lack empirical evidence.^7^^,^^6^ Since we believe that the only way to validate a quality model is to involve the target audience and the experts, the present qualitative study will combine the knowledge of educational experts and the needs of postgraduate learners.

The aim of this study is, firstly, to further investigate and clarify quality indicators of postgraduate medical e-learning by considering them from the perspective of e-learning experts and end-users. In this study, we will use the word “learners” to mean “end-users”. Secondly, it is to identify the deeper underlying foundation of these characteristics. This study will be, to our knowledge, the first to explore the needs of learners and the opinions of experts in postgraduate medical e-learning and will, therefore, aid in the definition of “good enough” e-learning. 

## Methods

### Study design

We decided to explore needs and opinions through focus-group discussions which were structured as per King’s template analysis. This qualitative approach aids the investigation of attitudes and beliefs, and also helps to generate new ideas and propel the development of theory.^10^ Larson and colleagues reported that an appropriate focus group is used for the following reasons: firstly, to gain clarity around people’s experiences of a given program; secondly, to generate information on participant attitudes and values; and thirdly, to add detail to previously generated information.^11^ We paid attention to the distinct difference between group discussion and group interview. The former is an in-depth discussion between participants, with the moderator only mediating to keep participants on-topic. In an interview, in contrast, the facilitator principally focuses on individual respondents in the group, rather than allowing or encouraging discussion between the respondents.^12^ The structure covered the domains from this review, and open questions were used to elicit in-depth and personal opinions, and ideas behind the domains. All the focus-group discussions started with the sharing of experiences of good and bad e-learning.

### Study participants

We used two groups of participants, as outlined in the current literature, namely, end-users of the e-learning programs (learners), and educators. It was considered that involving learners would ensure the e-learning programs became more learner-centred,^3^ while the educators could prevent a lack of learning strategies, assessment methods, and feedback mechanisms.^14^ We further divided the two groups, creating four groups in total. As research suggests that the upcoming generations (for example “generation Y”, born between 1980 and 1990) have different learning skills and needs,^15^ we divided the learners into two groups: experienced residents (born before 1980), and less experienced postgraduates (born after 1980). In our opinion, there are also two different groups of educators: those who are in the service of a university and/or teaching hospital, and those who are in the service of a commercial company. Therefore, we formed the following groups: more experienced learners (EU); less experienced learners (LU); methodological experts (educators) (ME); and commercial experts (CE). Citations in the results section will follow these abbreviations.

The more experienced and less experienced learners were invited in person to the teaching university hospital at the VU Medical Centre, Amsterdam, the Netherlands.  The methodological experts were educators invited from a national Dutch e-learning task group which we had contacted by e-mail and whose chairman put together a group of volunteers. The members of the commercial experts group were experienced designers and educators from a large e-learning company in the Netherlands, invited through the company director, who sent us an immediate response. The commercial educator group (CE) gave inputs based on their daily practice and years of designing experience, mainly from an educator’s point of view. A purposeful sampling was thus achieved to select the participants in the discussions. No financial compensation was given to any participant. The Dutch Association of Medical Education research gave ethical consent, after which all participants gave their written informed consent.

This study was conducted in the Netherlands, where residency training lasts between two and six years, depending on the discipline. Before that, the learners (EU and LU) had also received six years of undergraduate medical training. Medical educators belong to the Netherlands Association of Medical Education and have educating responsibilities at teaching hospitals and universities. E-learning is a well-developed area of commercial interest, with over 650 companies registered at the Chamber of Commerce in the Netherlands.

### Data collection method and procedure

To collect the data, we performed each focus-group discussion in the comfort of the participants’ own environment. The sessions were facilitated by RAL, lasted between forty-five and sixty minutes, and were audiotaped. We encouraged group discussion in which participants could dwell on the topics. Each new discussion allowed us to test and confirm the insights from the previous discussion. We continued conducting focus-group discussions until theoretical saturation was reached. The results of the discussions are presented using summaries and quotes.

### Data analysis

**Figure 1:**
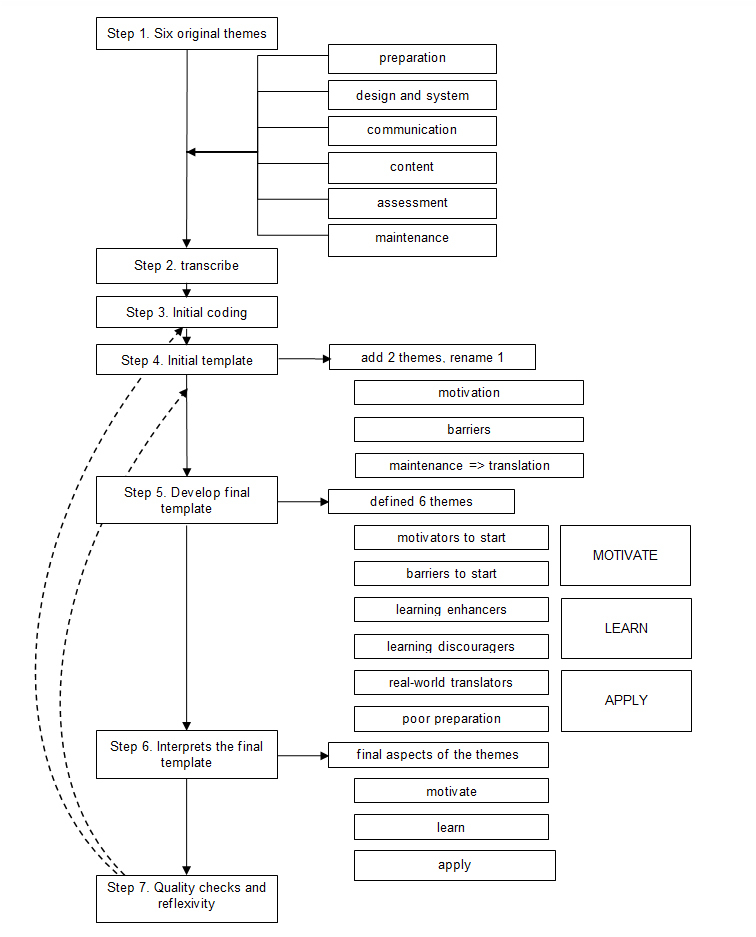
Seven steps of template analysis by King 2012

Our research question is to identify additional indicators to those mentioned in the previous literature review.^7^ For this reason, we analyzed the data of the focus-group discussions using King’s template analysis.^13^^,^^16^ This method requires seven steps: define an original template (for example, from a previous literature research); transcribe and code the discussions; structure these into an initial template; revise that template; generate the final template; interpret the final template; and perform a quality check (see Figure 1).

Our original template is based on the six themes derived from the previously identified Postgraduate Medical E-learning Model (ME Model),^7^^,^^13^ i.e., preparation, design and system, communication, content, assessment, and maintenance. The recordings were transcribed verbatim and entered into f4 analysis software using the software’s workflow.^17^ After transcribing the discussions (performed by RL to gain familiarity with the data), all data were coded. The data were then re-checked to find any overlap in codes, or the addition of any new ones. From the coding of the transcripts, we created thirty-four categories that were relevant for postgraduate e-learning. These items were sorted into the original template. Items that did not fit the template were grouped into new themes. FS and MW also read different parts of the transcripts and sorted the codes into the initial coding template. Emerging differences were discussed, and the template was altered as necessary. Further team discussions resulted in the final template, and full consensus was reached.

## Results

A total of four focus-group discussions, involving twenty-seven participants, took place between 1st November, 2015 and 7th April, 2016. The focus groups consisted of seven junior and six senior postgraduate learners, seven university education experts, and eight commercial educational experts. The mean age of the junior postgraduates and senior residents was 25 and 32, respectively. Overall, 88% of participants were female. During the fourth discussion, no additional categories, themes, or explanations emerged, after which information saturation was reached. Therefore, no new discussions were planned (Figure 1).

### The Template Analysis

During our analysis, several considerations emerged from the thirty-four categories (Table 1). Firstly, it was necessary to consider the dynamics of positives and negatives: some items should be included to make e-learning effective, while others should not. These positives and negatives can be regarded as being continuously weighed up by the learner: “Should I participate/continue, or should I stop”. Secondly, there was a perspective change. While the original template encompassed almost all items, it did not address the needs of more emotional and cognitive elements, such as feelings of urgency, stress, and doubt. The original template was focused on creating e-learning and all the steps needed for that. After combining design, communication, content, and assessment, two new themes were added (motivators and barriers). We ended up with six themes: three positives to include (motivation to start, learning enhancers, and real-world translators), and three negatives to avoid (barriers to starting, learning discouragers, and poor preparation).

**Table 1:** Template development from items to subjects

Items	Themes	Interpretation
Feeling of importance	Motivation to start	Motivation
Rewarding		
Defining the kind of e-learning		
Adding levels of learning		
Formulating learning objectives		
Visualizing learning goals		
Lack of anytime, anywhere availability	Barriers to starting	
Not convinced of the quality		
No added value		
Forced to start		
Not taken seriously		
Offering support	Learning enhancers	Learning
Horizontal communication		
Vertical communication		
Personalizing		
Problem-based learning		
Summarizing		
Repeating in different formats		
Providing feedback		
Using learning activities		
Activating and stimulating		
Offer knowledge overview		
Too long	Learning discouragers	
Too distracting		
Lacking user-friendliness		
No navigation overview		
Stressing learners		
Content not adapted		
Translating to the real world	Real-world translators	Applying
Using real-world examples		
Updating and maintaining		
Knowing your target audience		
Creating a development team	Preparation	
Planning a feasible budget		

### Theme One – Motivators to Start Learning

*“The purpose of the e-learning should be made clear… I want to know what I am about to learn”* (EU).

Both the learners and the educational experts agreed that the learner must be persuaded to start the e-learning. The first step was described by the educators as the most important: the learner should be motivated to pick up a device, or log in to the computer, and start the e-learning course. According to the educators, accessing intrinsic and/or eccentric motivation is essential.

**Figure 2:**
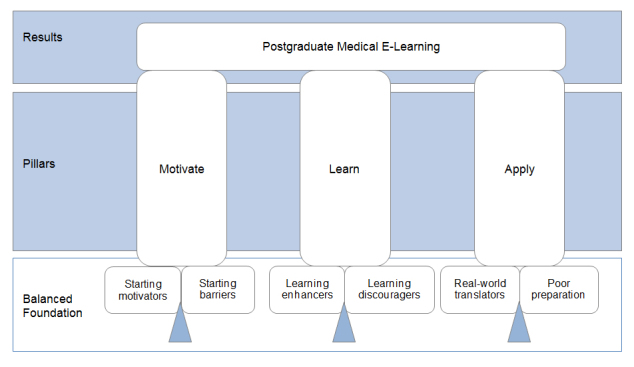
The final theme

To motivate the learner, the educator can use several methods, which were described by both end-users and educators. The experts appeared to believe that you should define the aim of e-learning (knowledge, skills, or behaviors/attitudes), formulate the learning objectives, and add levels of learning (the basic need-to-know level, a presumed-known lower level, and an in-depth higher level). The learners agreed with this analysis, even suggesting that the learning goals should be visualized:

*“The learning aim should be clear…the flow of the knowledge should be clear”* (EU).

*“You need to consider what has to be learned, how you know it has been learned...and which learning activities are needed...otherwise, you just get a lot of content”* (ME).

This quote is an example of the difference between a reference work and an educational tool. E-learning should motivate, because it is an educational tool.

*“The feeling of ownership, the feeling that you can control your learning path yourself, is motivating”* (CE).

Motivation can be created by a strong introduction, with an emphasis on the relevance of the topic and urgency (stimulating intrinsic motivation), but also by adding rewards or a deadline, or making the e-learning compulsory (eccentric motivation).

*“You must convince someone that the content is relevant for them”* (CE).

Although a learner can be motivated by the e-learning program, they might still not start if the barriers are too high, as illustrated by the next section.

### Theme Two: Barriers to Starting

By barriers, we mean the negatives that stand between a learner and the commencement of e-learning. When these barriers are too high, the learner might be motivated but still not start the e-learning program. One of the main benefits suggested by the learners and the e-learning experts is the ability to undertake the learning anytime and anywhere. The statements “Lack of access is a great barrier” (EU), and “E-learning is something I do when it suits my schedule” (EU) are examples of the way learners think about and experience e-learning. ‘Anytime, anywhere’ is, therefore, presumed to be important, and lack of access on these terms is a major barrier. Learners also agreed on another significant discouraging factor, namely doubt as to the quality of the e-learning program, and whether it offered added value over other didactic materials. This doubt can concern the educational quality:

*“I do not have a very positive attitude (towards) e-learning, because I don’t like the idea of just clicking through texts”* (LE);

or the content:

*“a textbook is always accurate and complete, but e-learning can miss out important things; therefore, I prefer the textbook when it’s important”* (LE).

If the learner is afraid that the e-learning course is incomplete, or when a traditional book provides more comfort in terms of quality, the learner would prefer to devote time to the traditional learning method. When the learner is in doubt, they experience “obligated e-learning” as being no longer motivating, but, rather, as a waste of their time:

*“A deadline motivates… but only when I am also convinced that the e-learning is actually teaching me something new” *(EU).

Aspects such as unrealistic deadlines, over-simple visuals or questions, and too much repetition with the same media cause the learner to feel they are not being taken seriously. Learners feeling that e-learning is not targeted at them, or doubting its quality, constitute barriers which may be too high.

After weighing the positives (motivators) and the negatives (barriers), it is hoped that the learner will start the e-learning program, which relates to the third and the fourth themes, as discussed below.

### Theme Three: Learning Enhancers

While engaged in e-learning, the learner is again weighing the positives and negatives. ‘Learning enhancers’ are understood to mean the positives experienced by the learner during the e-learning process. This theme includes items that motivate the learner to continue, but also the didactics which make learning (more) efficient. It is the combination of these motivators and didactics that keeps the learner focused on the e-learning program, and motivated to continue and finish it. The learners identify communication as an important part of the learning process. They want to be able to communicate between learners (horizontal communication), put questions to experts (vertical communication), and, in the event of technical problems, to receive prompt support. However,

*“(vertical) communication is only useful when you get a reply instantly”* (LE).

Educators emphasize the difference between graduates and postgraduates when considering the amount of freedom which should be granted to the learner. We called this “personalization”, by which we mean the option for a-linear learning: the ability to move from basic to advanced knowledge, and to skip those sections with which the learner is already familiar. “It's important to give freedom to the user to navigate through the content” (CE). Given the advances in problem-based learning (PBL), all educators and learners believe that this offers the best theoretical background.

*“The biggest benefit of problem-based learning...is the transfer to the workplace and therefore it is more motivating...especially for postgraduates”* (ME).

To maximize the learning experience, educators believe in summaries, repetition, provision of feedback, and stimulating the learner by interactivity.

*“The best way to repeat content is to wrap it in a different package every time. That way, you enable different learning styles (visual, textual, etc.) and the educator can repeat important lessons”* (CE).

During the whole process, the learner needs to be activated and stimulated by using different media. “It’s nice to have questions and feedback between the texts, which keeps me better focused than just sections of text would” (LE). However, learners also emphasized the value of different formats:

*“It was a short case-study, with a lot of visuals, and I had to move pictures around, which kept me busy. The content was a bit dull, but I really had the feeling I learned from it”* (LE).

At the end, the learner wants to be given an overview of the knowledge gained in order to know what they have learned. As the experts stated,

*“An educational tool is good when it enforces the lower-level students and raises their level of expertise”* (ME).

In regards to interactivity, the experts stated that

*“You want the learner to sit upright, not slouch”* (ME), and

*“You want to give a friendly poke in the ribs, without creating stress” *(ME).

During the learning process, the many issues which may discourage the learner are regarded as the negatives of the learning enhancers, as outlined below.

### Theme Four: Learning Discouragers

If the learning enhancers are minimal and the learning discouragers are multiplying, the learner might decide to stop before the learning aims are achieved. ‘Learning discouragers’ signifies those things that force the learner away from effective learning and may even drive them to stop. Both educators and learners gave examples of issues that can discourage to such an extent that the e-learning program is crippled. The learners identified these as a program that is too long, too distracting, lacks user-friendliness, fails to maintain learners’ interest, or even discourages them. The experts offered formulations such as

*“…a long e-learning program (which) obliges learners to study things they already know (and) generates irrelevant content”* (CE),

and stated that;

*“E-learning is not about transferring all the expert’s knowledge, but about transferring what is needed in the daily practice of the learner”* (CE).

There was some discussion about the ideal length of the e-learning course. Learners and educators say

*“Videos should not take much longer than 5 minutes... a module, a maximum of 20 to 30 minutes”* (LE),

and

*“… people’s attention can be held for 20 minutes … our modules are about 8 to 12 minutes each”* (ME);

and

*“If it takes too long, I just quit”*(LE).

They also emphasized the importance of a navigation overview to prevent learners becoming lost in the e-learning program. The educators tried to explain a paradox: how to motivate by offering deadlines and making the e-learning program mandatory, whilst not stressing the learner.

*“Stress…will overload your head and there will be no more room for learning” *(ME).

Therefore, stress can come from content which is too complicated or too simple, but also from unrealistic deadlines, or modules that last too long. Finally, the content should be adapted to the e-learning platform, and should be different from text-driven mediums like textbooks:

*“...when you only supply an online PowerPoint...you get a reference work and not an educational tool...there is a lack of interaction and, therefore, a lack of motivation” *(ME).

For example, sentences should be shorter and simpler.

When the learner finishes the e-learning program, the next step follows - real-world application. This is discussed in the next section.

### Theme Five: Real-World Translators

The purpose of e-learning is to use and apply new knowledge, skills, or behaviors in the real world. Therefore,

*“you need a continuous link with the daily practice of the user”* (CE).

‘Real-world translators’ is taken to mean the positives in this process of application, that is, those things in the e-learning program from which a learner needs to benefit most. The experts identified several items as important in translating e-learning to the real world. Firstly,

*“The learners need to be able to recognize themselves in the content, but also in the feedback and evaluation of the e-learning course”* (CE). 

An illustration of this would be using enough examples which the learner recognizes from their daily work:

*“When you work from a case-study or realistic situation, then you're more connected with the content and it's easier to remember and actually use the knowledge” *(EU). 

Secondly, e-learning can be kept connected to daily practice by being updated and maintained:

*“The design…like the font…should not look very old”* (LE). 

It is possible, and important, to keep e-learning current, and to designate the people who are responsible for doing so. The maintenance of an e-learning program entails ensuring it works on newly developed platforms, and incorporating feedback from learners.

There are, of course, also ways to stand in the way of translating the e-learning to the real world.

*“I believe a book is very limited and e-learning will be the future. You see it all around you”* (LE). 

When the learner cannot relate to the e-learning course, they may be motivated and even finish it, but will not use its content in their daily practice.

*“You need to know and define the needs and motivation of your target audience”* (CE). 

Both learners and educators frequently agreed that creators of e-learning must know their target audience, its current knowledge, education level, and the things it experiences at work.

*“If I give you a fourth-year science book, you will learn a lot less than an actual fourth-year student...that is because you need a reference framework” *(ME). 

If this consideration is not taken properly into account, learners cannot apply their e-learning.

### Theme Six: Poor Preparation

It might seem strange to finish with the theme of preparation; however, poor preparation can cause harm to both real-world translation and the continuation of e-learning.  Therefore, one must start with proper preparation, even if the effects of poor preparation might only manifest themselves at the end of the e-learning process.

‘Poor preparation’ is taken to mean that the e-learning program is neither created properly, nor given the attention it deserves during the creation. Two important elements of the preparation are the budget and team collaboration.

*“Bad e-learning is e-learning created from one perspective without the collaboration of the target audience, content experts, education experts, and ICT staff”* (CE). 

An education expert helps because they

*“will answer the questions of what the learning aims are, and how you know these have been acquired”* (ME). 

Secondly, cost-planning must be borne in mind, and creators must be aware that profits may be small or non-existent:

*“I would never pay for e-learning, because once you have done it, it’s useless”* (LE). 

To prevent this, the educators suggested creating a dedicated development team, and careful planning of costs.

### Final template interpretation

According to King’s template analysis, after the final template has been created there is room for its interpretation. Above, we have created a final template containing six themes - three positives and three negatives. From a consideration of the positives and negatives, we have distilled three aspects that are positively and negatively influenced: motivate, learn, and apply (Figure 2). This is a learner-centered construct of subjects in chronological order. In Figure 2, we visualize the process of first, motivating (balancing the motivational items and barriers); then the learning itself (balancing learning enhancers and discouragers); and, finally, the application of the e-learning content (which is only possible when it can be translated to the real world, that is, when it has not been hampered by poor preparation).

## Discussions

We believe the final template confers added value on the original template. After combining some of the original themes, we lifted them to a higher level of cognition and evaluation, which are what the learner uses to weigh up the e-learning process. We ended with six categories: three positives to be included (motivation to start, learning enhancers, and real-world translators) and three negatives to be avoided (barriers to starting, learning discouragers and poor preparation). We believe that the only way to find these underlying constructs is by holding focus-group discussions. We did not find enough arguments to limit the final template to the three aspects of motivate, learn, and apply. If the template were limited to these three alone, it would lose the necessary nuances. The added value conferred by the addition of the final themes is in regard to learner-centeredness. We also believe creating broad themes, gives more freedom in the e-learning design, and more focus on the real needs. One example is the Design and System domain from the original template. One of the items is *“Use software depending upon flexibility”. *

This may seem straightforward; however, it does not touch on the real issue. We prefer: Does your e-learning have any barriers to starting? As a designer will have an example of these barriers, namely poor, inflexible software. However, this formulation forces an educator to think about other barriers, or even accept one barrier (poor software, for example, because they have no other options) but compensate with a powerful motivator (for example, a free mobile device which runs that software).

Another example is the content domain, one of the items in this case being the use of cognitive multimedia principles. The underlying need, however, is *for “learning enhancers”*: which particular aspects the educator chooses to use to improve or enhance learning does not matter. Our previous literature study showed, however, that the cognitive load principle is a particularly valuable example.

Notions from existing literature underpin the identified model. Schumacher and colleagues, for instance, describe several learning and motivational theories to construct three subjects which are analogous to our motivate, learn, and apply: the desire to learn; the ability to learn; and a context and environment for learning.^18^ Motivation has been identified as the essential component that stimulates and sustains learning behavior. While motivation may not be the main stimulus for learning, it definitely plays a critical role during the learning process.^19^ Some studies have considered individual items such as rewards (the positive aspects of rewards are to be utilized)^20^, while others give examples of themes such as barriers to e-learning.^21^ In a recent study, Reid and colleagues described several obstacles to e-learning engagement in medical students.^22^ There is an interesting overlap in the obstacles found in their study and the negatives of the template described in the current paper. Reid and colleagues described the feeling of unfairness, which is a barrier comparable to the barrier to starting in our ‘motivation’ subject. The feeling identified by Reid and colleagues of being lost and overwhelmed could create a learning distraction and disengagement, which might be a result of poor preparation and poor user connection. The overlap between the obstacles found by Reid and the negative themes of the present study suggest, perhaps, a general working model in which different focus discussions from different research groups arrive at a similar conclusion.

### Generic or postgraduate-specific model

As with the template model, there are very few specific items for medical postgraduates. One could argue that motivate, learn, and apply is a mantra for all (adult) learning; however, there is a difference in the specifics. As the educators state:

*“The postgraduate needs to be able to scroll through subjects that are neither too simple, too boring, nor irrelevant, so he or she has the freedom needed to remain motivated” *(CE).

This consideration is because

*“postgraduates value flexibility more than graduates, and have more self-discipline”* (ME). 

Their motivation is also different; for example,

*“for a medical student, a good e-learning program is one that prepares you for an exam; for a postgraduate, it is only good when it's relevant for their daily practice” *(ME). 

Furthermore,

*“postgraduate e-learning should be more focused on skills and learning tasks than on plain knowledge”* (ME). 

Researchers have applied different theories of adult learning and, although debatable, recent meta-analysis has even suggested that motivation and learning changes with age.^23^ The template may be partly generic, but others have come to a similar conclusion in the competency-based framework towards which medical education is growing, in which (postgraduate) learners drive their own education.

### Limitations

Certain limitations must be acknowledged. The very nature of this method means that it may not always lead to the same conclusions. The interpretations of the codes, the template, and the final subjects might be influenced by cultural values, earlier experience, and prejudice concerning the original template. The authors have used the discussions described and the international literature to broaden their view and make the model as generalized as possible. Two limitations are specific to this study, apart from the difficult distinction between generic characteristics and postgraduate specifics.

The first of these limitations is the range of items extracted from the discussions. The learners are limited by their experience, and the educators are limited by their view of medical education. Therefore, we tried to extract the underlying constructs and use the items as examples, rather than as the only possible items.

Secondly, the definition of e-learning is broad and culturally diverse,^24^ and learners and educators seem to diverge in their understanding of the meaning of the term. While learners have a clear idea about e-learning - *“just learning with the computer” *(LU) - educators believe that there are many different forms of e-learning, with different goals. E-learning is learning through a digital medium, but there are great differences between blended learning, hybrid learning, simulation, serious gaming, and others. These activities also have different learning goals, which may be the acquisition of new knowledge, skills, attitudes or behaviors. Our proposed themes could fit all these e-learning variations and goals. The specifics within the themes are key to the variations in e-learning, goals, and target audience.

## Conclusions

As noted above, low-fidelity, high-quality e-learning could be a major education disruptor; however, we need more insight into the characteristics and indicators of quality. This is the first qualitative study, to our knowledge, to incorporate the perceptions of postgraduate end-users of the quality of their e-learning. We have raised a postgraduate medical e-learning quality model to a higher level by defining the actual needs and constructs behind the detail. We concluded that three negative and three positive themes are balanced by the learner, and could be responsible for the essence of good (enough) postgraduate medical e-learning. Further research should be carried out to validate these themes, for example by asking learners to evaluate their e-learning using this model.

### Acknowledgements

We wish to thank our participating postgraduates, the medical educators, and TinQwise for their time, effort, and honest inputs in the focus-group discussions. Furthermore, we wish to thank Mr. Kieran Walsh for his constructive feedback and Mr. Sha’er Ramkalup for his help with the discussions and transcripts. Finally, we wish to thank Ms. Rees for correcting the English of the draft manuscript. There was no financial support for this study.

### Conflict of Interest

The authors declare that they have no conflict of interest.
